# Case Report: Challenges in diagnosing Kawasaki disease in children under 3 months of age: a case series report

**DOI:** 10.3389/fped.2025.1490921

**Published:** 2025-07-08

**Authors:** Duc Long Phi, Thi Hoai Nguyen, Khanh Linh Duong, Cam Anh Nguyen Le, Duy Cuong Nguyen, Van Thuan Hoang

**Affiliations:** ^1^ Thai Binh University of Medicine and Pharmacy, Thai Binh, Vietnam; ^2^ Thai Binh Pediatric Hospital, Thai Binh, Vietnam

**Keywords:** Kawasaki, infant, coronary aneurism, dual antiplatelet drug therapy, echocardiogram (ECHO)

## Abstract

Infants younger than 3 months old often present with incomplete Kawasaki Disease (KD), where not all the classical features are present. This makes the diagnosis challenging, as KD may be easily confused with other common pediatric conditions, such as viral infections, bacterial sepsis, or toxic shock syndrome. Moreover, the risk of developing coronary artery abnormalities is reportedly higher in younger infants, making timely diagnosis and treatment critical. Here we reported three cases of KD in infants under three months, each illustrating the challenges in diagnosis due to the absence of typical KD symptoms such as rash, conjunctivitis, and oral mucosal changes. Echocardiography played a pivotal role in identifying coronary artery abnormalities, leading to the diagnosis of incomplete KD in all cases. Clinicians should maintain a high index of suspicion for KD in any febrile infant, particularly when inflammatory markers are elevated. Early recognition and treatment are vital to prevent severe cardiovascular complications.

## Introduction

Kawasaki disease (KD), first described by Dr. Tomisaku Kawasaki in 1967 (1), is an acute, systemic vasculitis that primarily affects children under five years old. The exact etiology of KD remains unknown, but it is believed to involve an abnormal immune response to an infectious or environmental trigger in genetically predisposed individuals. KD is the leading cause of acquired heart disease in children in developed countries, with coronary artery aneurysms being the most severe complication if left untreated (2).

The clinical diagnosis of KD is typically based on the presence of prolonged fever (lasting at least five days) and at least four of the following five principal clinical features: bilateral non-exudative conjunctivitis, changes in the lips and oral cavity, polymorphous rash, changes in the extremities (such as erythema of the palms and soles, or desquamation), and cervical lymphadenopathy (3). However, these criteria were established based on children older than 6 months, and their application in infants, particularly those under three months of age, can be problematic (4, 5). The 2024 American Heart Association guidelines have updated the diagnostic criteria for Kawasaki Disease, notably reducing the required fever duration from at least five days to four, especially in cases with strong supportive features such as coronary abnormalities. This change has important implications for early diagnosis in infants, who often present with incomplete criteria and rapid coronary involvement (6).

Infants in this age group often present with incomplete KD, where not all the classical features are present (4, 5). This makes the diagnosis challenging, as KD may be easily confused with other common pediatric conditions, such as viral infections, bacterial sepsis, or toxic shock syndrome. Moreover, the risk of developing coronary artery abnormalities is reportedly higher in younger infants, making timely diagnosis and treatment critical (2, 7).

Given these challenges, this case series aims to highlight the diagnostic difficulties encountered in infants under three months of age with KD. Through the presentation of three cases, we aim to underscore the importance of considering KD in the differential diagnosis of febrile infants, even in the absence of the full complement of clinical signs, and the critical role of echocardiography in early detection and management.

## Case presentation

Case 1: A 2-month-old male infant presented with a high fever and cough on the second day of illness. Clinical examination showed no other abnormalities. Initial laboratory tests revealed leukocytosis with a white blood cell (WBC) count of 15.23 G/L and an elevated C-reactive protein (CRP) level of 52.14 mg/dl (Table 1). Rapid tests for influenza and SARS-CoV-2 were negative. Despite treatment with intravenous third-generation Cephalosporin antibiotics for a presumed diagnosis of sepsis, the fever persisted after five days. Notably, the child lacked other symptoms typically associated with KD, such as rash, conjunctivitis, or changes in the oral mucosa.

**Table 1:** Laboratory findings of patients.

Laboratory findings	Case 1		Case 2		Case 3	
	Admission Day 2 of illness	Day 5 of illness	Admission Day 2 of illness	Day 5 of illness	First hospitalization	Second hospitalization
White blood cell (10⁹/L)	15.23	17.8	9.9	14.3	13.23	11.15
Neutrophil (10⁹/L)	8.8	8	5.4	9.2	5.56	2.49
Lymphocyte (10⁹/L)	5.5	7.8	3.3	3.2	4.42	7.68
Red blood cell (10¹²/L)	3.52	2.91	4.59	4.45	2.79	5.16
Hemoglobin (g/L)	106	86	120	111	82	112
Platelets (10⁹/L)	445	708	441	521	655	369
CRP (mg/L)	52.14	295.44	8.2	80.12	33.77	43.33
Ure (mmol/L)	2.42	1.44	2.96	2.9	-	-
Creatinine (µmol/L)	35.1	30.5	36.5	34.1	-	-
AST (U/L)	36.3	27.3	71.6	125.2	30	35.9
ALT (U/L)	25.4	12.4	49.1	115.7	14	14.6

**Figure 1:**
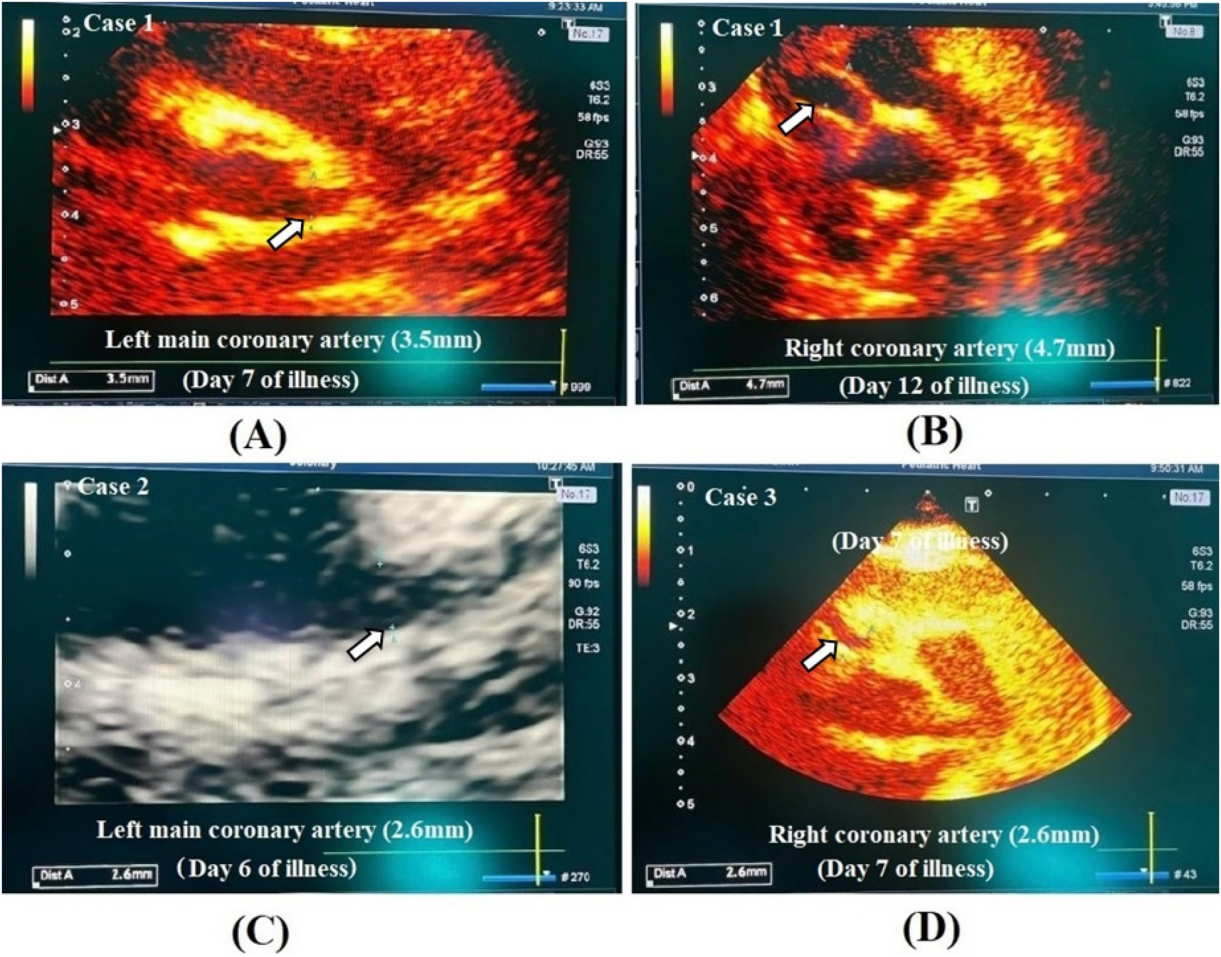
Echocardiographic image of coronary artery lesion in case 1 **(A,B)**, case 2 **(C)** and case 3 **(D)**.

On the fifth day of illness, laboratory tests showed a further increase in WBC count to 24.88 G/L and CRP to 233.75 mg/dl (Table 1). Regarding the persistent fever and elevated inflammatory markers, a transthoracic echocardiogram was indicated at day 7 of illness and revealed significant coronary artery dilation, with a left coronary artery diameter of 3.5 mm (z-score 5.86), left anterior descending coronary artery (LAD) of 1.9 mm (z-score 2.43), left circumflex coronary artery (LCX) of 1.7 mm (z-score 2.09), right coronary artery diameter of 2.4 mm (z-score 3.86), and a subsequent segment of 3.8 mm (z-score 7.24) (Figure 1A). The diagnosis of incomplete KD was made, and the child received intravenous immunoglobulin (IVIG) and aspirin. The fever resolved 21 hours after IVIG administration. A follow-up echocardiogram at day 12 of illness showed worsening coronary artery dilation with a left coronary artery diameter of 3.3 mm (z-score 5.39), left anterior descending coronary artery (LAD) of 2.8 mm (z-score 5.3), right coronary artery diameter of 4.4 mm (z-score 10.64) (Figure 1B). The patient was transferred to the National Children's Hospital for further treatment with anticoagulation and dual antiplatelet therapy. Finally, he was eventually discharged in stable condition.

Case 2: This case involved a girl of three months old who had been treated for pneumonia at a district hospital and had been discharged seven days prior to presentation. She developed high fever, vomiting, and loose stools two days before admission. Laboratory tests showed a WBC count of 9.9 G/L and a CRP level of 8.2 mg/dl (Table 1). Despite ongoing high fever, the child exhibited only mild symptoms, such as red, dry lips and a slightly red tongue, without rash, conjunctivitis, or lymphadenopathy.

Due to persistent high fever with limited clinical findings and mildly elevated inflammatory markers (Table 1), an echocardiogram was obtained at day 6 of illness to rule out cardiovascular involvement. This revealed mild coronary artery dilation, with a left coronary artery diameter of 2.6 mm (z-score 2.99), LAD of 1.6 mm (z-score 0.96), LCX of 1.4 mm (z-score 0.78), and right coronary artery diameter of 1.9 mm (z-score 1.85) (Figure 1C). The child was diagnosed with incomplete KD and initially received empiric antibiotics for suspected infection while awaiting diagnostic clarification. Upon confirmation of coronary involvement, aspirin therapy was initiated as part of KD management. The fever resolved within three days, and the child was discharged in stable condition with a maintenance dose of aspirin.

Case 3: A 32-day-old boy presented with severe respiratory symptoms, including a hoarse cough, wheezing, rapid breathing, and a high fever. The child also had a mottled skin rash, red sclera, and red, cracked oral mucosa. Laboratory tests indicated a WBC count of 13.23 G/L and a CRP level of 33.77 mg/dl (Table 1). The echocardiogram did not show coronary artery dilation at the time. The child was treated for severe pneumonia and discharged after 10 days.

However, at four months of age, he was readmitted with a recurrent pneumonia. Upon readmission, because of persistent inflammation (Table 1), an echocardiogram was performed at day 7 of illness to evaluate possible complications, including KD-related coronary involvement. This showed a significant coronary artery dilation, with a left coronary artery diameter of 2.6 mm (z-score 3.89), LAD of 2.7 mm (z-score 4.76), LCX of 1.5 mm (z-score 1.4), and right coronary artery diameter of 4.1 mm (z-score 9.34) (Figure 1D). The child was retrospectively diagnosed with KD, which had been missed during the initial hospitalization. The child received antibiotic therapy for presumed respiratory infection, but following retrospective diagnosis of KD based on echocardiographic findings, aspirin was introduced to address coronary artery abnormalities, and he was discharged.

## Discussion

Diagnosing Kawasaki disease in infants under three months of age is particularly challenging due to the nonspecific and often subtle clinical presentation (4, 5, 8). In children older than 6 months, the diagnosis of KD is primarily clinical, based on the presence of fever and a characteristic set of symptoms (9). However, in infants, especially those under three months, the presentation is frequently atypical, leading to delayed diagnosis and treatment, which can result in severe complications, particularly coronary artery aneurysms (4, 10–12).

As demonstrated by our cases, children under three months of age with KD may initially present with prolonged fever as the only apparent symptom. Other classical signs, such as rash, conjunctivitis, or changes in the oral mucosa, may be absent or appear late in the disease course. This incomplete presentation can easily lead to misdiagnosis or a delay in considering KD as a differential diagnosis, especially in a setting where infectious causes of fever are more common and initially prioritized. Several studies (5, 13) have emphasized that infants under 3 months are at particularly high risk for missed or delayed KD diagnosis. Current American Heart Association guidelines recommend initiating evaluation for incomplete KD when inflammatory markers such as C-reactive protein (CRP) exceed 30 mg/L or erythrocyte sedimentation rate (ESR) exceeds 40 mm/h, particularly when these values persist beyond 3–4 days in the absence of an identifiable infection, even when few or no principal clinical features are present (14). This approach is especially important in young infants, who often lack the full clinical constellation of KD.

In case 1, the initial diagnosis was sepsis due to fever and respiratory symptoms, with antibiotics given empirically. It was only after several days of persistent fever and a marked increase in inflammatory markers that KD was considered. The absence of classic symptoms such as rash, red eyes, and oral changes further complicated the diagnosis (15). This delay in recognizing KD highlights the need for clinicians to maintain a high index of suspicion for KD in febrile infants, even when typical signs are not present.

Echocardiography is a critical tool in the diagnosis and management of KD, particularly in atypical cases (16). Coronary artery involvement, manifesting as dilation or aneurysms, is the most severe complication of KD and can be present even in the absence of other clinical features. The detection of coronary artery abnormalities on echocardiography can confirm the diagnosis of KD in cases where clinical signs are ambiguous or incomplete (5, 16). In these cases, echocardiography was not routine but was prompted by persistent fever unresponsive to antibiotics and elevated inflammatory markers. This diagnostic approach aligns with recommendations to consider echocardiography in febrile infants when clinical suspicion of KD exists despite an incomplete presentation.

In case 2, the infant exhibited only mild symptoms, yet echocardiography revealed coronary artery dilation, leading to the diagnosis of incomplete KD. This underscores the importance of performing an echocardiogram in any infant with unexplained prolonged fever, especially when inflammatory markers such as CRP are elevated. Early identification of coronary artery involvement allows for timely initiation of IVIG therapy, which is crucial in preventing the progression of coronary artery abnormalities (17).

The early diagnosis of KD in young infants is crucial, as delayed treatment increases the risk of coronary artery aneurysms. Studies have shown that infants under six months are more likely to present with incomplete KD and are at higher risk for coronary artery involvement (4, 8, 10–13). This increased risk, coupled with the diagnostic challenges, necessitates a heightened awareness among clinicians.

The third case illustrates the consequences of a missed diagnosis. The infant initially presented with severe respiratory symptoms and a widespread rash, which was attributed to a respiratory infection. The diagnosis of KD was missed, and it was only during a subsequent hospitalization that coronary artery dilation was detected on echocardiography. This case emphasizes the importance of considering KD in the differential diagnosis of any infant with prolonged fever, even when other causes appear more likely (18). Kawasaki disease is typically self-limiting; however, without appropriate treatment such as IVIG, patients are at increased risk of developing coronary artery complications. This case highlights that although the fever was resolved, the underlying vasculitis progressed, as evidenced by coronary dilation on follow-up.

Indeed, in case 1, although IVIG therapy led to fever resolution within 21 h, follow-up echocardiography showed progression of coronary artery dilation. Similarly, in case 3, the initial echocardiogram during the first hospitalization was normal, despite the infant presenting at least four principal features of KD including rash, conjunctival injection, and changes in the oral mucosa. The later development of coronary artery aneurysms upon readmission strongly suggests that subclinical or smoldering inflammation persisted beyond the acute phase. This raises the possibility that inflammation may not have been fully controlled despite initial clinical improvement. Incomplete suppression of vascular inflammation, sometimes referred to as smoldering or subclinical vasculitis, has been documented in patients with KD and may contribute to worsening coronary artery outcomes even after timely IVIG treatment. These cases highlight a critical consideration in infants under three months: the physical manifestations of inflammation may be subtle or transient, and inflammatory activity can continue despite apparent clinical improvement. Therefore, serial echocardiographic follow-up is essential in this age group, even when initial imaging appears normal. Such an approach allows for early detection of evolving coronary pathology and timely adjustment of management strategies to mitigate long-term cardiovascular risks (6, 17).

While traditional inflammatory markers such as CRP and ESR remain valuable in KD diagnosis, newer biomarkers and diagnostic algorithms have also been investigated (6). Biomarkers like NT-pro BNP, D-dimer, and markers of fibrinolysis may support early KD diagnosis and predict coronary complications. However, their utility in infants under three months is limited. For example, NT-pro BNP levels are physiologically elevated in neonates and infants due to postnatal cardiovascular adaptation, which reduces their specificity for KD in this age group. Despite these limitations, integrating biomarker trends with clinical findings and echocardiography may improve diagnostic accuracy in ambiguous cases (6, 9, 17).

The cases presented in this series highlight several important considerations for clinicians managing febrile infants. First, KD should be considered in any infant with prolonged fever, particularly when the fever is accompanied by elevated inflammatory markers, even if the classic signs are absent. Second, echocardiography should be performed early in the diagnostic workup of suspected KD, as coronary artery abnormalities may be the first or only indication of the disease in this age group. Finally, prompt initiation of treatment with IVIG and aspirin is essential to reduce the risk of coronary artery aneurysms and other complications.

Given the diagnostic challenges associated with KD in infants under three months, there is a need for greater awareness and consideration of atypical presentations. Early recognition and intervention can significantly improve outcomes, reducing the risk of long-term cardiovascular complications and improving overall prognosis (Box 1).

BOX 1 **Clinical red flags for early Kawasaki disease evaluation in infants <3 months.**

**Table:** 

Clinical finding	Implication
Persistent fever ≥4 days	Consider incomplete KD even without full clinical features
CRP >30 mg/L and/or ESR >40 mm/h	Marker of significant inflammation per AHA criteria
No response to empiric antibiotics	Suggests non-bacterial etiology; supports KD suspicion
Unexplained sterile pyuria, irritability, or hepatomegaly	Nonspecific signs that may indicate systemic vasculitis
→ Prompt echocardiography recommended	Especially if inflammatory markers elevated

## Conclusion

While diagnostic difficulties in very young infants with KD are well recognized, there remains a paucity of case-based data from low- and middle-income settings, particularly Southeast Asia. Our series contributes additional insight into how incomplete presentations can delay diagnosis and lead to adverse coronary outcomes, underscoring the need for improved awareness and adapted diagnostic strategies. Indeed, this case series underscores the importance of considering KD in the differential diagnosis of febrile infants, even when classical signs are absent. Echocardiography plays a critical role in identifying coronary artery involvement, which is a key indicator of KD in this age group. Early diagnosis and prompt treatment with IVIG and aspirin are crucial to prevent severe complications such as coronary artery aneurysms. Clinicians should maintain a high index of suspicion for KD in young infants with prolonged fever, and further research is needed to develop better diagnostic strategies for this vulnerable population.

## Data Availability

The original contributions presented in the study are included in the article/Supplementary Material, further inquiries can be directed to the corresponding author.
